# Hybrid versus conventional endoscopic submucosal dissection for treatment of gastric lesions: a comparative systematic review and meta-analysis

**DOI:** 10.1016/j.igie.2023.03.007

**Published:** 2023-06-07

**Authors:** Thomas R. McCarty, Ahmad Najdat Bazarbashi, Michael Senter-Zapata, Russell D. Dolan, Christopher C. Thompson, Hiroyuki Aihara

**Affiliations:** 1 Lynda K. and David M. Underwood Center for Digestive Disorders, Houston Methodist Hospital, Houston, Texas, USA; 2 Division of Gastroenterology and Hepatology, School of Medicine, Washington University, St Louis, Missouri, USA; 3 Department of Internal Medicine, Brigham and Women's Hospital, Harvard Medical School, Boston, Massachusetts, USA; 4 Division of Gastroenterology, Hepatology and Endoscopy, Brigham and Women's Hospital, Harvard Medical School, Boston, Massachusetts, USA

## Abstract

**Background and Aims:**

Hybrid endoscopic submucosal dissection (ESD) may overcome the complexity of conventional ESD associated with gastric lesions. The aim of this study was to perform a systematic review and meta-analysis to compare the efficacy and safety of hybrid versus conventional ESD for the treatment of gastric lesions.

**Methods:**

Individualized search strategies were developed in accordance with Preferred Reporting Items for Systematic Reviews and Meta-Analyses and Meta-Analysis of Observational Studies in Epidemiology guidelines. Pooled proportions were calculated with rates estimated using random-effects models. Measured outcomes included en-bloc resection, procedure-associated adverse events, and procedure duration. Heterogeneity was assessed with the *I*^2^ statistic and publication bias using funnel plots and Egger regression testing.

**Results:**

Of 5 included comparator studies (hybrid ESD, 184 patients; conventional ESD, 289 patients), 1 was a randomized trial and 4 were retrospective observational studies. Mean patient age was 68.89 ± 4.75 years, and the average lesion size was 17.81 ± 5.58 mm. Hybrid ESD patients were older (*P* < .001) with smaller lesions (15.75 ± 4.72 mm vs 19.12 ± 5.69 mm; *P* < .001). Overall, the en-bloc resection rate was significantly decreased for hybrid ESD (odds ratio [OR], .11; 95% confidence interval [CI], .02-.62; *P* = .010). Total adverse events were not different between groups (OR, 1.56; 95% CI, .44-5.53; *P* = .490). Rates of delayed bleeding (OR, 1.47; 95% CI, .34-6.40; *P* = .610) and perforation (OR, 2.41; 95% CI, .65-9.12; *P* = .194) were also not significantly different. Procedure time was significantly shorter for hybrid ESD (mean difference, 15.13 minutes; 95% CI, 4.05-26.21; *P* = .007).

**Conclusions:**

Although hybrid ESD for gastric lesions was associated with significantly shorter procedure times compared with conventional ESD, hybrid ESD was associated with lower rates of en-bloc resection and similar adverse events.

Since the 1980s, EMR has been a well-established endoscopic treatment strategy for the removal of early, superficial gastric malignancies.^1^^,^^2^ EMR, a simplistic approach that uses a cautery-enhanced electrosurgical snare device to resect target tissue in an en-bloc or piecemeal fashion, has predominated in the West. On the other hand, endoscopic submucosal dissection (ESD) was developed in Japan in the 1990s as a first-line method for treating superficial malignancies of the GI tract.^3^ ESD traditionally involves a circumferential incision followed by submucosal dissection using an electrosurgical knife. Whereas EMR relies on piecemeal resection for lesions >15 to 20 mm and has higher disease recurrence rates, ESD enables en-bloc resection regardless of size or presence of submucosal fibrosis and has lower disease recurrence rates. Removing an entire lesion en bloc by conventional ESD allows for accurate histologic assessment and staging, determination of margin negativity, and results in very low disease recurrence.^4^ Despite these advantages in ESD, widespread adoption in the United States has not occurred because of procedural complexity, concern for adverse events, procedure duration, and lack of reimbursement models.

Multiple studies have compared EMR with ESD for the treatment of superficial gastric lesions, finding ESD to provide superior resection outcomes but with an increased risk of adverse events.^5^, ^6^, ^7^ Given the current landscape of conventional ESD with its associated advantages and disadvantages, hybrid ESD has been developed with a primary goal to decrease procedural complexity and incorporate the benefits of both ESD and EMR.^8^ Hybrid ESD combines submucosal injection, initial circumferential incision, and partial submucosal dissection with snaring to excise target lesions, which in theory may decrease operative time, adverse event rates, and overall procedure difficulty. Although ESD has been shown repeatedly to be associated with improved rates of complete and curative resection compared with piecemeal EMR, comparative data of conventional ESD versus hybrid ESD remain limited.^9^ As such, we aimed to perform a structured systematic review and meta-analysis of all available published data to evaluate the efficacy and safety of hybrid versus conventional ESD for the treatment of gastric lesions.

## Methods

### Literature search

Individualized literature search strategies were performed to identify relevant articles comparing conventional versus hybrid ESD. Systematic searches of PubMed, EMBASE, Web of Science, and the Cochrane Library databases were performed from the available literature from inception through December 31, 2022 using the following medical subject heading terms: “hybrid endoscopic submucosal dissection (ESD) OR snare endoscopic submucosal dissection (ESD).” Related articles were subsequently reviewed by subject heading search terms, title, and abstract for “gastric OR stomach.” Subject heading search terms and title and abstract were then reviewed.

All relevant English language full-text articles regardless of year of publication were included in this systematic review and meta-analysis. From the initial search results, duplicate articles were extracted, and then the titles and abstracts of all potentially relevant studies were screened for eligibility. The reference lists of studies of interest were then manually reviewed for additional articles by cross-checking bibliographies. Two reviewers (T.R.M. and A.N.B.) independently screened titles and abstracts of all articles according to predefined inclusion and exclusion criteria. Any differences were resolved by mutual agreement and in consultation with a third reviewer (R.D.D.). For studies with incomplete information, contact was attempted with the principal authors to obtain additional data.

### Study registration and literature search

This study was prospectively submitted in PROSPERO, an international database of prospectively registered systematic reviews in health and social care. The Preferred Reporting Items for Systematic Reviews and Meta-Analyses statement outline and Meta-Analysis of Observational Studies in Epidemiology reporting guidelines for reporting systematic reviews and meta-analyses were used to report findings (Appendices 1 and 2, available online at www.igiejournal.org).^10^^,^^11^

### Inclusion and exclusion criteria

Full-text articles were included in this analysis with abstracts (oral presentation and published) excluded. Only direct comparator studies of hybrid versus conventional ESD for gastric lesions in adult patients were included. Noncomparator studies or articles evaluating nonhybrid or alternative endoscopic techniques (ie, precut EMR) were excluded. Hybrid ESD was defined by circumferential incision and partial submucosal dissection followed by resection with the use of a snare. Conventional ESD was defined by circumferential incision followed by submucosal dissection without the use of a snare device. Studies deemed to have insufficient data, review articles, editorials, and correspondence letters that did not report independent data were also excluded. Case series and reported studies with <10 patients were excluded to minimize selection bias. Multiple published work from similar authors was evaluated for overlapping enrollment times to preserve independence of observations.

### Outcome measures

The primary outcome measurements in this study were the efficacy and safety of hybrid ESD versus conventional ESD for the treatment of superficial gastric lesions. Efficacy and safety of hybrid ESD were measured by pooled en-bloc resection rates, procedure-associated adverse events, and rates of recurrence. Adverse events were stratified by type of event, including perforation or delayed bleeding. Secondary outcomes of interest were procedure duration (measured in minutes) and local recurrence rates. Secondary reported characteristics were baseline patient and procedure characteristics including gastric lesion size and location and lesion type.

### Risk of bias and quality assessment

Risk of bias and quality for randomized trials was performed using the JADAD score and observational studies were evaluated using the Newcastle-Ottawa Quality Assessment Scale.^12^^,^^13^ In this study, high quality was defined for randomized studies by a JADAD score ≥3 and a Newcastle-Ottawa Quality Assessment Scale score ≥4. Two authors (T.R.M. and A.N.B.) independently extracted data and assessed the risk of bias and study quality for each article. Any disagreements were resolved by discussion and consensus and in consultation with a third reviewer (R.D.D.).

### Investigations of heterogeneity

Heterogeneity was assessed for the individual meta-analyses using the χ^2^ test and the *I*^2^ statistic.^14^ Significant heterogeneity was defined as *P* < .05 using the Cochran Q test or *I*^2^ > 50%, with values >50% indicating substantial heterogeneity. Further quantification of heterogeneity was categorized based on *I*^2^ with values of 25%, 50%, and 75% indicating low, moderate, and high amounts of heterogeneity, respectively.

### Publication bias

A funnel plot was created and visually inspected for asymmetry and quantitatively used Egger regression testing to assess for publication bias.^15^^,^^16^ If evidence of publication bias was found, then the trim and fill method was used to correct for funnel plot asymmetry and provide an adjusted effect.^17^

### Statistical analysis

This systematic review and meta-analysis was performed by calculating pooled proportions. After appropriate studies were identified through systematic review, the individual study proportion was transformed into a quantity using the Freeman-Tukey variant of the arcsine square root–transformed proportion. The pooled proportion was then calculated as the back transform of the weighted mean of the transformed proportions and DerSimonian-Laird weights for the random-effects model.^18^^,^^19^ The pooled rates were estimated using random-effects models and presented as point estimates (rates) with 95% confidence intervals (CIs).^14^^,^^20^^,^^21^

Statistical significance for the differences between groups included the 95% CIs of the 2 pooled proportions considered, and the differences of proportions and 95% CIs were calculated. To evaluate differences in procedure duration between hybrid and conventional ESD, weighted mean difference was calculated and transformed to the natural logarithm before pooling, and the variance was calculated. All calculated *P* values were 2-sided, and *P* < .05 was considered statistically significant. Tabular and graphic analyses were performed using Comprehensive Meta-Analysis software, version 3 (BioStat, Englewood, NJ, USA). Combined weighted proportions were determined by use of the Stata 15.0 software package (Stata Corp LP, College Station, Tex, USA).

## Results

### Included studies

**Figure 1:**
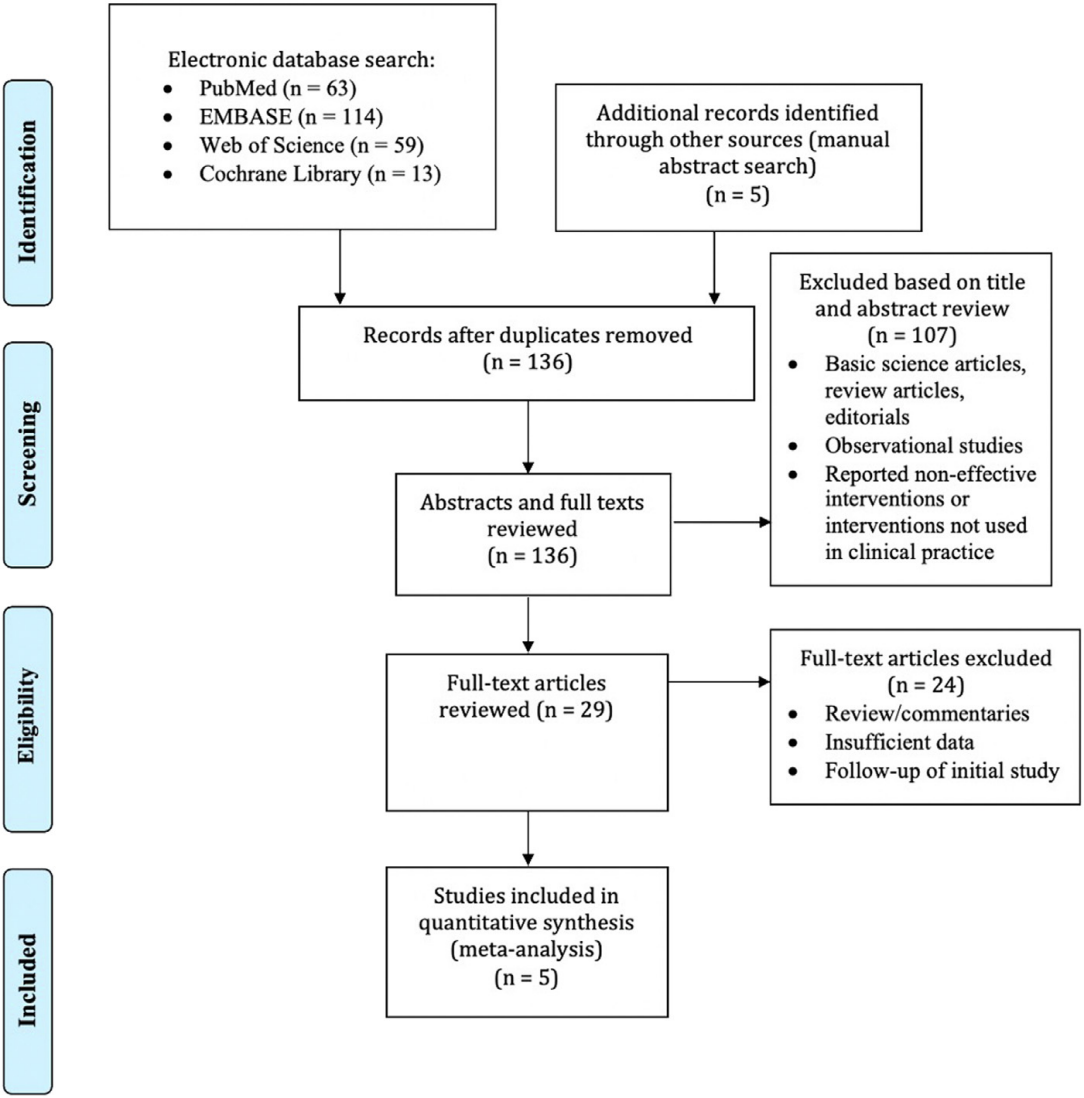
Preferred Reporting Items for Systematic Reviews and Meta-Analyses flowchart of the literature search results for gastric lesions.

Five studies (473 patients) were included in this meta-analysis.^22^, ^23^, ^24^, ^25^, ^26^ Of the 473 patients, 289 patients were included in the conventional ESD cohort and 184 patients in the hybrid ESD group. A Preferred Reporting Items for Systematic Reviews and Meta-Analyses flowchart of the search results is shown in Figure 1. Studies were published from 2009 to 2022, with 1 multicenter randomized controlled trial and 4 retrospective studies included. Among the retrospective studies, 3 single-center studies and 1 multicenter study were included. The studies were from Japan and Korea. Hybrid versus conventional ESD data are summarized in Table 1.

**Table 1:** Baseline characteristics of included studies assessing hybrid vs conventional endoscopic submucosal dissection

Study	Year	Country	Design	Type	No. of patients	Mean age (y)	No. of men	Lesion type	Mean lesion size (mm)	Histology	En-bloc resection rate	Adverse event rate	Delayed bleeding rate	Perforation rate	Procedure time (min)	Study quality
*Hybrid endoscopic submucosal dissection*																
Esaki et al^26^	2022	Japan	Multicenter, randomized controlled trial	Full text	39	76	24	Elevated (25), flat (2), depressed (14)	10	—	39/39	1/39	0/39	0/39	33.16	3∗
Esaki et al^22^	2020	Japan	Retrospective, multicenter (propensity-score matched)	Full text	29	77	19	Elevated (12), flat/depressed (17)	10	—	29/29	0/29	0/29	0/29	20	6
Kim et al^23^	2018	Korea	Retrospective, single center	Full text	26	64	16	Elevated (14), flat (7), depressed (5)	23	Gastritis (3), LGD (17), HGD (3), CA (3)	25/26	0/26	0/26	0/26	9.7	5.5
Jang et al^24^	2014	Korea	Retrospective, single center	Full text	45	65.2	30	Elevated (45)	18.2	LGD (20), HGD (25)	41/45	4/45	4/45	0/45	19.9	5.5
Goto et al^25^	2009	Japan	Retrospective, single center	Full text	45	68.5	37	—	17.8	—	41/45	8/45	5/45	3/45	70.2	6
*Conventional endoscopic submucosal dissection*																
Esaki et al^26^	2022	Japan	Multicenter, randomized controlled trial	Full text	40	76	25	Elevated (23), flat (0), depressed (17), mixed (1)	9	—	40/40	2/40	2/40	0/40	62.46	3∗
Esaki et al^22^	2020	Japan	Retrospective, multicenter (propensity-score matched)	Full text	29	72	18	Elevated (19), flat/depressed (10)	10	—	29/29	0/29	1/29	0/29	40	6
Kim et al^23^	2018	Korea	Retrospective, single center	Full text	12	60.8	8	Elevated (11), flat (1), depressed (0)	28	Gastritis (2), LGD (8), HGD (1), CA (1)	12/12	0/12	0/12	0/12	12.8	5.5
Jang et al^24^	2014	Korea	Retrospective, single center	Full text	54	62.3	39	Elevated (54)	19.6	LGD (19), HGD (35)	54/54	4/54	3/54	1/54	33.8	5.5
Goto et al^25^	2009	Japan	Retrospective, single center	Full text	154	68.1	118	—	22.6	—	154/154	6/154	3/154	3/154	75.8	6

*LGD*, Low-grade dysplasia; *HGD*, high-grade dysplasia; *CA*, cancer.

∗ JADAD score used for the randomized controlled trial. All other quality scores were evaluated using the Newcastle-Ottawa Quality Assessment Scale as retrospective, observational studies.

### Patient and gastric lesions characteristics

Mean patient age was 68.89 ± 4.75 years, and hybrid ESD patients were older compared with conventional ESD patients (69.99 ± 5.17 years vs 68.20 ± 4.33 years, *P* < .001). Gender was similar between hybrid versus conventional ESD (68.48% men vs 71.97% men, *P* = .417). All lesions included in this meta-analysis were adenomas or early-stage cancer. Individual lesion types and histopathologic diagnoses are shown in Table 1. The average gastric lesion size included in this study was 17.81 ± 5.58 mm. Hybrid ESD patients had smaller lesions (15.75 ± 4.72 mm vs 19.12 ± 5.69 mm, *P* < .001) compared with conventional ESD patients.

### Study quality and risk of bias assessment

**Figure 2:**
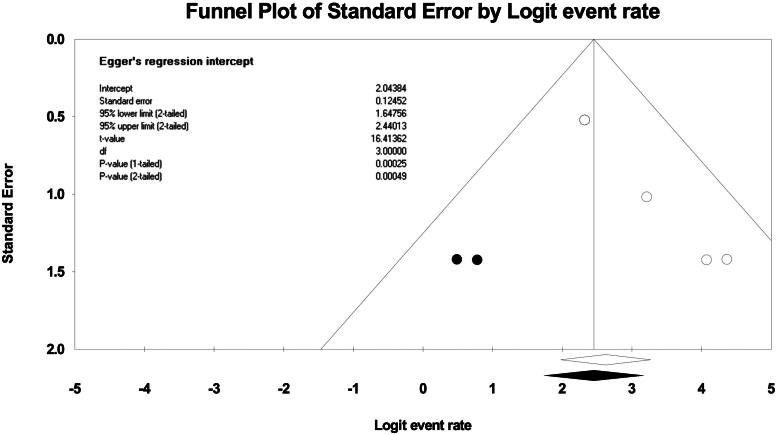
Funnel plot of publication bias and Eggers regression test for included studies.

Quality assessment for each study was determined to be of high quality (Newcastle-Ottawa Quality Assessment Scale scores ≥4 and JADAD score ≥3) as demonstrated in Table 1. Publication bias was also assessed. Visual inspection of funnel plots demonstrated that smaller and statistically insignificant studies appeared to be missing likely because of publication bias (Fig. 2). With the Duval and Tweedie’s trim and fill method, overall en-bloc resection of hybrid and conventional ESD was not significantly different because of the presence of overlapping CIs. Egger regression testing did not reveal evidence of publication bias.

### Primary and secondary comparator outcomes

**Figure 3:**
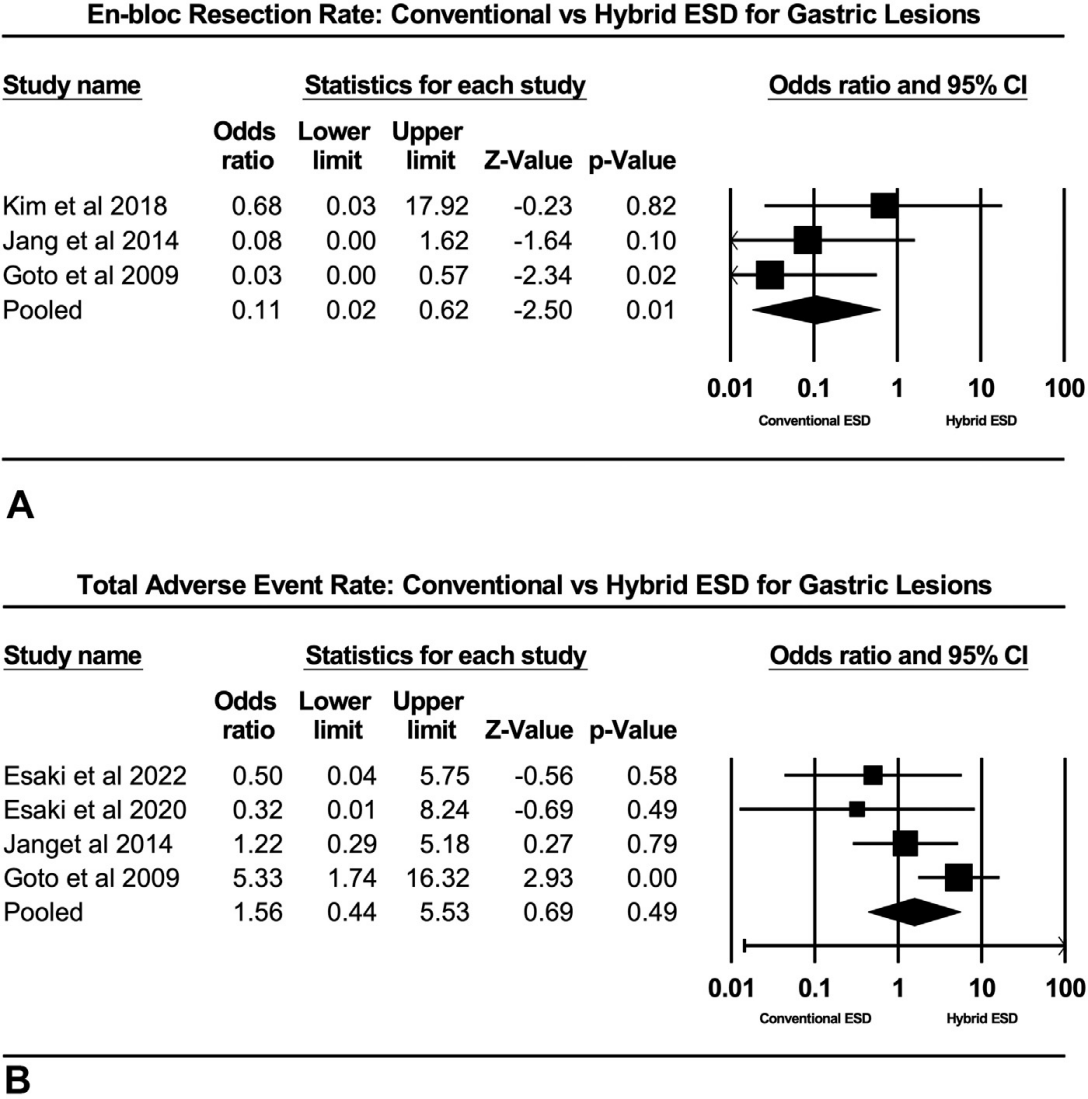
**A,** Rate of en-bloc resection: hybrid endoscopic submucosal dissection (ESD) versus conventional ESD. **B,** Rate of adverse events: hybrid ESD versus conventional ESD. *CI*, Confidence interval.

**Figure 4:**
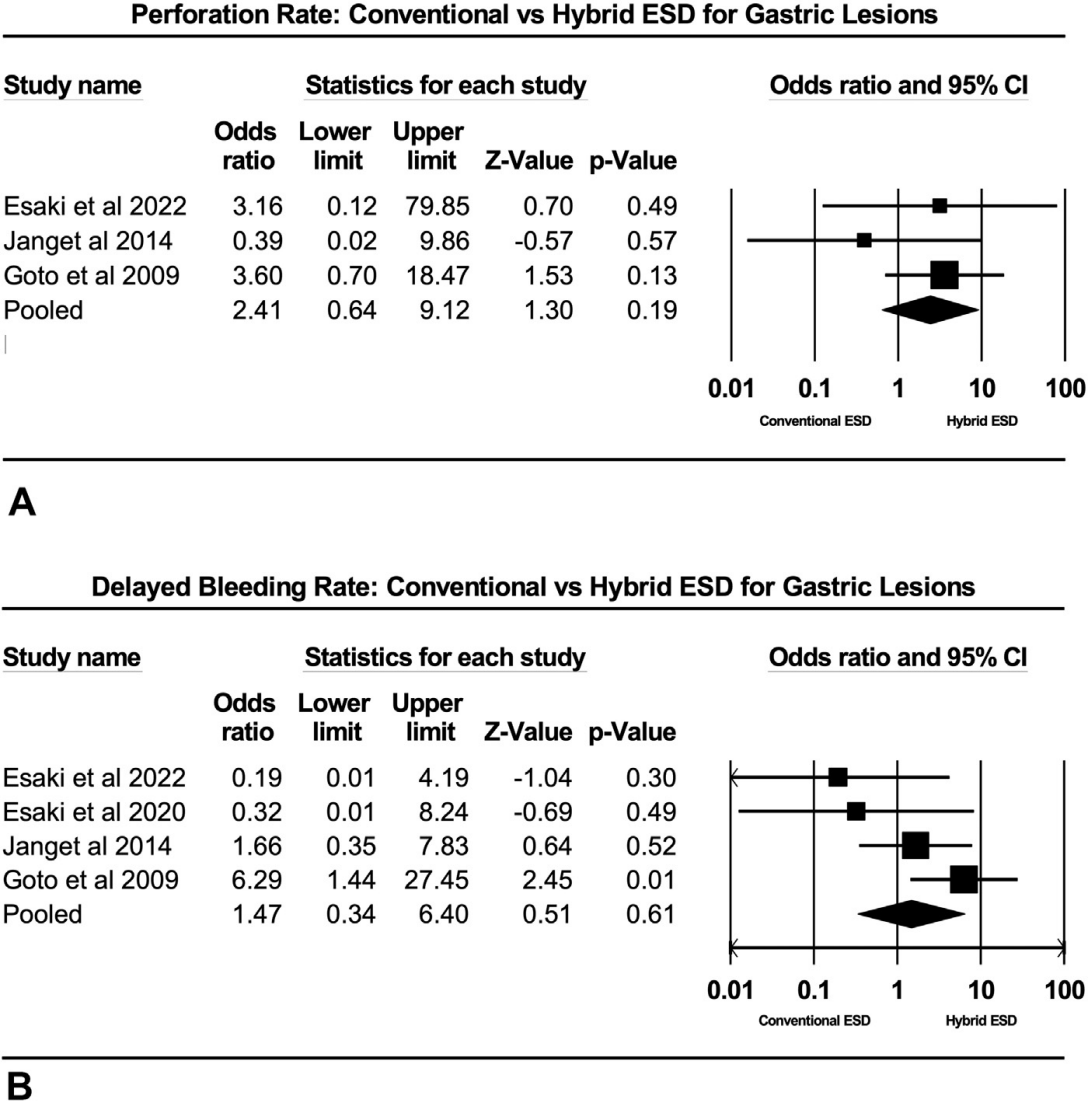
**A,** Rate of delayed GI bleeding: hybrid endoscopic submucosal dissection (ESD) versus conventional ESD. **B,** Rate of perforation: hybrid ESD versus conventional ESD. *CI*, Confidence interval.

In terms of procedural efficacy, summary characteristics for each dissection strategy are highlighted in Table 2. Based on all included studies, the en-bloc resection rate was significantly decreased for hybrid ESD compared with conventional ESD (odds ratio [OR], .11; 95% CI, .02-.62; *P* = .010, *I*^2^ = .00%) (Fig. 3A). Total adverse events were not different between groups (OR, 1.56; 95% CI, .44-5.53; *P* = .490, *I*^2^ = 48.49%) (Fig. 3B). Next, adverse events were stratified by type of adverse event. There was no difference in perforation rate (OR, 2.41; 95% CI, .65-9.12; *P* = .194, *I*^2^ = 30.69%) or delayed GI hemorrhage (OR, 1.47; 95% CI, .34-6.40; *P* = .610, *I*^2^ = 48.35%) between hybrid and conventional ESD (Fig. 4A and B). Only 1 study reported local recurrence, so this could not be analyzed. Procedure time was significantly shorter for hybrid ESD (mean difference, 15.13 minutes; 95% CI, 4.05-26.21; *P* = .007, *I*^2^ = 97.66%).

**Table 2:** Procedure-associated outcomes of hybrid vs conventional endoscopic submucosal dissection

Outcomes	Hybrid endoscopic submucosal dissection	Conventional endoscopic submucosal dissection
En-bloc resection rate	93.23 (87.84-96.33) *I*^2^ = .00	98.83 (96.02-99.66) *I*^2^ = .00
Overall adverse event rate	7.01 (2.74-16.80) *I*^2^ = 51.45	4.83 (2.85-8.07) *I*^2^ = .00
Rate of perforation	3.70 (1.61-8.28) *I*^2^ = .00	1.94 (.08-4.41) *I*^2^ = .00
Rate of delayed bleeding	6.83 (3.27-13.72) *I*^2^ = 18.45	3.79 (2.02-7.02) *I*^2^ = .00

Values are % (95% confidence interval).

## Discussion

Based on the results of this systematic review and meta-analysis, hybrid ESD is associated with a significantly shorter procedure duration. Despite this decreased time to complete gastric lesion resection, hybrid ESD was associated with a lower en-bloc resection rate, despite smaller lesions in the hybrid ESD group. Additionally, there was no improvement in safety profile (ie, comparable rate of adverse events) between hybrid and conventional ESD. Stratifying these types of adverse events further by perforation or delayed GI bleeding also revealed no difference between the hybrid and conventional ESD techniques.

Before comparing hybrid versus conventional ESD, it is critical to understand what type of gastric lesions are amenable to endoscopic resection. According to the most recent Japanese Gastric Cancer Association guidelines, the absolute indications for ESD or EMR include a differentiated-type adenocarcinoma without ulcerative findings in which the depth of invasion is clinically diagnosed as T1a and the diameter of the lesion is ≤20 mm.^27^ In a meta-analysis comparing outcomes of ESD versus EMR including over 4300 lesions, conventional ESD was associated with a higher complete en-bloc resection rate (OR, 9.7; 95% CI, 7.7-12.1) and complete histologic resection rate (OR, 5.7; 95% CI, 2.9-11.0).^5^ Local recurrence, an outcome this meta-analysis was unable to analyze, was significantly decreased in the ESD cohort as well (OR, .09; 95% CI, .05-.17); however, there was a higher risk of perforation (OR, 4.7; 95% CI, 2.8-7.9) with no difference in rate of delayed GI bleeding.

Hybrid ESD is characterized by partial submucosal dissection followed by snare-assisted resection, seeking to incorporate strategies of both ESD and EMR, in effort to obtain optimal results. Although size is believed to remain a limiting factor with traditional EMR, the ability to perform dissection may improve the rate of en-bloc resection (avoiding piecemeal resection). However, the results of this systematic review and meta-analysis demonstrate that the en-bloc resection rate was significantly decreased for hybrid ESD compared with conventional ESD, despite the fact that patients who underwent hybrid ESD had smaller gastric lesions. Interestingly, there was no difference in adverse events between resection techniques, although the hybrid ESD procedure duration was significantly decreased compared with conventional ESD.

More recently, our group performed a similar systematic review and meta-analysis of the hybrid ESD technique for colorectal lesions.^4^ In that study, we found, similar to this study, that hybrid ESD was associated with significantly shorter procedure duration compared with conventional ESD. However, unlike this study, hybrid ESD appeared to be associated with fewer adverse events for colorectal lesions. However, no difference was found in adverse events for gastric lesions, likely related to location of the lesions, because traditionally colorectal ESD is considered more difficult than gastric ESD.^3^^,^^28^ Therefore, hybrid ESD may be more effective in the colon as compared with gastric lesions, potentially because of the technical difficulty and a higher chance of adverse events in colorectal ESD; however, more data are needed to validate this statement.

This study is not without limitations. Importantly, despite these studies being comparative in nature, pooled analyses revealed these patient populations were not similar. Additionally, given the retrospective nature of included studies, which may lead to selection bias and result in further heterogeneity of our results, these results should be interpreted with some caution. Additional heterogeneity was a result of differences in patient age, endoscopic strategy, snare devices, needle-knives used for dissection, endoscopic experience, lesion location, and histology. Although 1 study was a propensity score–matched analysis, designed to overcome many of these limitations, differences and heterogeneity among pooled results persisted. Ultimately, well-designed, randomized studies to control for measured and unmeasured confounders are needed to better evaluate these various dissection techniques.

Despite these limitations, this study possesses several strengths. To our knowledge, this is the first systematic review and meta-analysis to assess the efficacy and safety of hybrid versus conventional ESD for upper GI lesions. Furthermore, although data are limited and are largely based on retrospective literature, this study represents an important step in establishing future algorithms or endoscopic treatment recommendations. Although further randomized trials would provide much-needed information, these will be difficult to conduct, especially in the West where the hybrid technique may be more widely used as a rescue technique for incomplete or difficult to perform ESD.

In summary, hybrid ESD has a comparable safety profile and significantly decreased procedure time when compared with conventional ESD for removal of gastric lesions. However, despite this comparable safety profile, hybrid ESD is associated with a lower rate of en-bloc resection. Future studies to investigate outcomes of hybrid ESD versus piecemeal EMR may also be highly relevant to endoscopists in the West.

## Disclosure

*The following authors disclosed financial relationships: C. C. Thompson: Consultant for Apollo Endosurgery, Boston Scientific, Covidien/Medtronic, Fractyl, GI Dynamics, Olympus/Spiration, and USGI Medical; research* *support* *from* *Apollo Endosurgery**,* *Aspire Bariatrics**,* *GI Dynamics**, Olympus/Spiration, Spatz, and USGI Medical; general partner in BlueFlame Healthcare Venture* *Fund; board member with EnVision Endoscopy, Fractyl, and USGI Medical; ownership interest in GI Windows. H. Aihara: Consultant for Olympus America, Boston Scientific, Fujifilm Medical Systems, Auris Health, Lumendi, Medtronic, ConMed, and 3D Matrix. All other authors disclosed no financial relationships.*

## Supplementary data

PRISMA Checklist

MOOSE Checklist
